# 8-OXO-Cordycepin Is Not a Suitable Substrate for Adenosine Deaminase-Preliminary Experimental and Theoretical Studies

**DOI:** 10.3390/molecules30163377

**Published:** 2025-08-14

**Authors:** Boleslaw T. Karwowski

**Affiliations:** Nucleic Acids Damage Laboratory, Faculty of Pharmacy, Medical University of Lodz, ul. Muszynskiego 1, 90-151 Lodz, Poland; boleslaw.karwowski@umed.lodz.pl

**Keywords:** cordycepin, adenosine deaminase, 7,8-dihydro-8-oxo-3′deoxyadenosine, DFTB

## Abstract

Adenosine deaminase (ADA) is one of the most important enzymes in nucleoside metabolism, regulating the levels of adenosine and deoxyadenosine triphosphate (ADT/dATP) on either side of the cell membrane. This small protein (weighing approximately 40 kDa) exhibits deamination properties towards other pharmaceuticals built on adenine as the leading structure, which requires co-administration of ADA inhibitors. 3′-deoxyadenosine (Cordycepin, Cord) is an active compound isolated from the fungus *Cordyceps*, which has been used in traditional Chinese medicine for over 2000 years. Its anticancer activity is likely related to the inhibition of primer elongation of lagging strands during genetic information replication. Unfortunately, Cord is rapidly deaminated by ADA into inactive 3′-deoxyinosine, necessitating its co-administration with ADA inhibitors. Here, for the first time, the synthesis and discussion of the oxidised form of Cord are presented. The 7,8-dihydro-8-oxo-3′-deoxyadenosine (Cord^OXO^) exhibits high resistance to ADA because of its *syn* conformation, as shown experimentally by UV spectroscopy and RP-HPLC monitoring. Theoretical Density Functional based Tight Binding (DFTB) studies of the Michaelis complex ADA-Cord^OXO^ have revealed significant distance increases between the “active” H_2_O molecule and C6 of the 8-oxo-adenine moiety of Cord^OXO^, i.e., 4 Å as opposed to 2.7 Å in the cases of ADA-dAdo and Cord. In conclusion, it can be postulated that the conversion of Cord to Cord^OXO^ enhances its therapeutic potential; however, this needs to be verified in vitro and in vivo. It should be emphasised that the therapeutic effect, if any, can be achieved theoretically without ADA inhibitors, e.g., pentostatin, thus reducing adverse effects. These promising preliminary results, presented here, warrant further investigations.

## 1. Introduction

**Figure 1:**
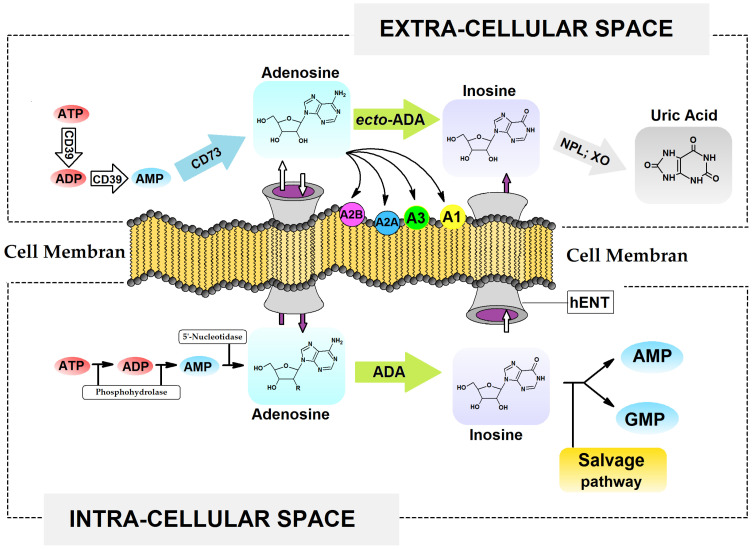
Overview of extracellular and intracellular adenine nucleotide signalling, metabolism, and transport. **A1**, **A2A**, **A2B**, **A3** Adenosine receptors, **ATP**, **ADP**, **AMP** Adenosine Tri- Di- Mono- Phosphat, **GMP** guanosine monophosphate, **ADA** Adenosine DeAminase, **XO** Xantine Oxidase, **NPL** Nucleoside PhosphoryLase, **CD39** ectonucleoside-triphosphate-diphosphohydrolase 1, **CD73** ecto-59-nucleotidase, **hENT** Human Equilibrative Nucleoside Transporter.

Nucleosides and their phosphorylated derivatives (Figure 1) present in the extracellular space regulate various processes, including neurotransmission, angiogenesis, immune response, and vasodilation [1]. Their activity depends on various factors, including ATP release, specific receptor binding, metabolism, and uptake, and their interactions with the G-protein-coupled receptors P2X and P2Y [2]. Enzymes such as CD39 (Ectonucleoside triphosphate dephosphohydrolase-1), CD73 (ecto-5′-nucleotidase), and ADA (adenosine deaminase) regulate the intra- and extracellular levels of adenosine nucleotides (e.g., AMP, ATP, cAMP), which interact with various G-protein-coupled receptors, e.g., A1, A2B, A2A, and A3 [3]. It should be noted that the physiological concentrations of dNTP, such as ATP, dATP, and the dIno pool, are replenished either by *de novo* or salvage pathways, depending on the microenvironmental conditions [4,5] (Figure 1). Consequently, their therapeutic “target” potential in terms of their molecular structures and functions has been extensively investigated for decades [6]. Therefore, the role of adenosine derivatives in tumourigenesis is highly significant and cannot be underestimated. Adenine nucleosides are a “response” to several hallmarks of cancer, such as rapid proliferation, immortality, evasion of the immune system, unregulated replication, angiogenesis, and metastasis [2,7]. Numerous compounds have been obtained and tested in preclinical studies, and some are in the clinical development stage [8]. Furthermore, new therapies (gapmer, siRNA, CRISPR/Cas9, and antisense oligonucleotides) based on the dogma that nucleic acids carry genetic information essential for life processes, such as growth, development, and reproduction, are becoming the cornerstones of modern pharmacology [9]. The last three decades have seen the development of extensive diagnostic techniques and the implementation of personal health awareness programmes, alongside a worrying increase in the consumption of ultra-processed foods. These trends are reflected in the rising cancer incidence, as revealed by global statistical analyses [10,11,12]. The limited efficacy of existing therapies has forced scientists from different fields to search for new, safer, and more effective medical treatments.

Much attention has been paid to one such alternative: the *Cordyceps* fungus *Cordyceps militaris* (L.) *Fr*, *Cordyceps sinensis*, which has been used in traditional Chinese medicine for hundreds of years. The main active compound has been identified as the adenosine analogue, Cordycepine (3′-deoxyadenosine, Cord) [13]. It has a wide range of physiological functions, including anti-inflammatory, analgesic, immune-modulating, antioxidant, antiviral, anticancer, and anti-metastatic effects [14]. The latter two pharmacological effects are related to polyadenylation inhibition, which affects cell replication and maturation [15]. It should be noted that, unfortunately, the half-life of Cord measured in vivo (using rats as a model) is relatively short, i.e., t_1/2_ = 1.6 min [16].

From a structural and chemical perspective, cordycepin is a twin analogue of two canonical nucleosides: adenosine (Ado) and 2′-deoxyadenosine (dAdo). This makes it a convenient substrate for Adenosine DeAminase (ADA, EC 3.5.4.4) [17]. As mentioned, ADA is an important protein that regulates the concentration of ATP/dATP in the extracellular and intracellular environments. ADA (metalloprotein aminohydrolase) is present on both sides of the cell membrane and catalyzes the conversion of Ado and dAdo to inosine (Ino) and 2′-deoxyinosine (dIno), respectively [18]. As expected, ADA metabolises and deactivates Cord to 3′-deoxyInosine (3′-dIno) in a highly effective manner [18,19]. Given the importance of the level of ATP in the microenvironment of lymphocyte T/B surfaces for the immune response, ADA plays a vital role in maintaining homeostasis, and its deactivation, inhibition, or deficiency causes haematologic malignancies [20]. Therefore, from a therapeutic and pharmacological point of view, extending the half-life of Cord in the presence of both isoforms of ADA, i.e., ADA1 and ADA2, is of utmost importance. Although some selective inhibitors, such as 2′-deoxycoformycin (2′-dCof, pentostatin) and erythro-9-(2-hydroxy-3-nonyl) adenine (EHNA), have been identified, their co-administration with Cord causes undesirable side effects related to considerable increases in ATP/dATP concentration levels [21,22]. Furthermore, all active compounds administered to the human body are continuously exposed to various physiological conditions that can change their structure and therapeutic properties. Because of the similarity of Cord to canonical adenine nucleosides, its conversion to 7,8-dihydro-8-oxo-3′-deoxyadenosine (Cord^OXO^) is expected [23]. In this article, for the first time, the resistance of Cord^OXO^ to reactions catalysed by adenosine deaminase is investigated and discussed alongside its synthesis, both experimentally and theoretically.

## 2. Results

### 2.1. Chemical Synthesis of 7,8-Dihydro-8-Oxo-3′-Deoxyadenosine and Its Characterisation

**Figure 2:**
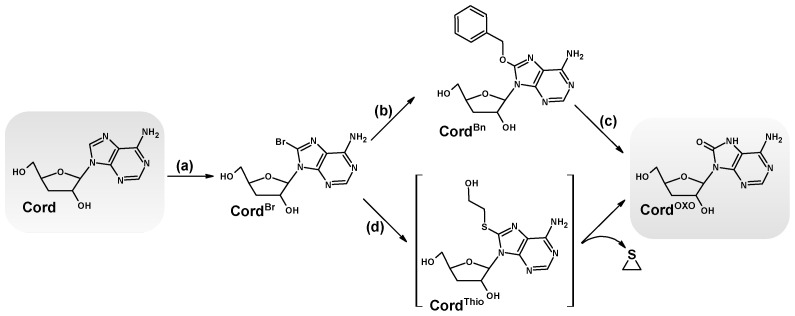
Scheme of two possible synthesis pathways of 7,8-dihydro-8-oxo-3′-deoxyadenosine: (**a**) aqua bromide, H_2_O, (**b**) benzyl alcohol, DMSO, Na, (**c**) 10% Pd/C, methanol, (**d**) triethylamine, 2-mercaptoethanol, H_2_O.

**Figure 3:**
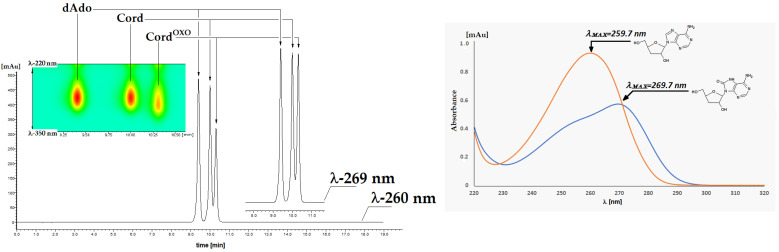
RP-HPLC chromatogram with DAD detection of the dAdo (10.13 min), Cord (9.53 min), Cord^OXO^ (10.43 min) mixture, recorded in the range of 220–350 nm. The differences in UV spectra profiles between Cord and Cord^OXO^ were *λ_MAX_*-269.7 nm and *λ_MAX_*-259.7 nm.

As mentioned in the introduction, Cord is an analogue of canonical dAdo and Ado. Thus, it seems reasonable to expect that this purine nucleoside analogue will be subject to the same oxidation and free radical processes, leading to the formation of 7,8-dihydro-8-oxo-3′-deoxyadenosine (Cord^OXO^) [23]. Cord^OXO^ was synthesised for the first time according to the typical synthetic methodology used for 8-oxo-purine synthesis [24]. As shown in Figure 2, the starting substrate Cord was halogenated by aqueous bromide to yield 8-bromo-3′-deoxyadnosine (Cord^Br^) with a 90% yield. Cord^Br^ was subsequently converted by the reaction with 2-mercaptoethanol to the final product Cord^OXO^ with excellent efficiency (80%), according to the method described by Navacchia et al. [25]. An alternative method based on Williamson ether synthesis was investigated and performed [26]. 8-Benzyloxy-3′-deoxyadenosine, without chromatographic purification after 10% Pd/C hydrogenolysis, resulted in the final product Cord^OXO,^ yielding 40%. However, it was not used further due to its lengthy reaction time and low synthesis yield. ^1^H NMR spectral analysis revealed the characteristic cordycepin shift of the 2′ and 3′ protons, comparable to dAdo. Moreover, the UV spectra of Cord^OXO^ showed maximum absorbance at λ = 269.7 nm, while for Cord, it was measured at λ = 260 nm, which is typical for canonical nucleosides (Figure 3). Moreover, the UV spectral profile was found to be similar to that of 7,8-dihydro-8-oxo-2′-deoxyguanosine (dG^OXO^). Chromatographic analysis of Cord^OXO^ mobility on reverse-phase support using HPLC showed a longer elution time than the parent Cord and dAdo, i.e., 10.43, 10.13, and 9.53 min, respectively (Figure 3). These results indicate an increase in the lipophilicity of cordycepin after its oxidation to Cord^OXO^ derivatives.

### 2.2. The Stability of 7,8-Dihydro-8-Oxo-3′-Deoxyadenosine in the Presence of Adenosine Deaminase

The levels of adenosine nucleotides, i.e., ATP and dATP, in the extra-and intracellular environments are regulated by adenosine deaminase, which converts adenine to hypoxanthine via a hydrolysis reaction. The same function and chemistry have been observed in therapeutic compounds built on adenine as the primary structure [27,28].

**Figure 4:**
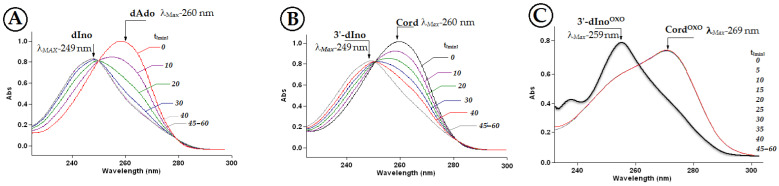
UV spectra of deamination reactions catalysed by adenosine deaminase of (**A**) dAdo → dIno, (**B**) Cord → 3′-dIno, (**C**) Cord^OXO^ → non-product with the maximum of absorption indicating product and substrate obtained during a period of 60 min with five-minute intervals at 21 °C, the black solid curve represents the requested UV spectra of 3′-dIno^OXO^.

Therefore, the stability of Cord^OXO^ in the presence of ADA was investigated and compared with that of Cord and dAdo. The deamination reaction was performed in 50 mM HEPES buffer (pH 7.3) at 21 °C. The reaction progress was first investigated for dAdo and Cord by absorption measurements in the range of 220–300 nm. The initial maximum was exhausted at λ_Max_ = 260 nm and disappeared, and a new maximum appeared at λ_Max_ = 249 nm, which corresponded to dIno. As shown in Figure 4A,B, during one hour, the successive maximum corresponding to substrate exhaustion was observed, with an increase in the subsequent maximum of the product. Surprisingly, in a parallel experiment, Cord^OXO^ was resistant to adenosine deaminase activity (Figure 4C), and an unexpected maximum of 259 nm was observed (Figure 4C). The above results were confirmed by sets of corresponding experiments under RP-HPLC control every 60 min with detection in the range of 190–310 nm using a Diode Array Detector (DAD).

**Figure 5:**
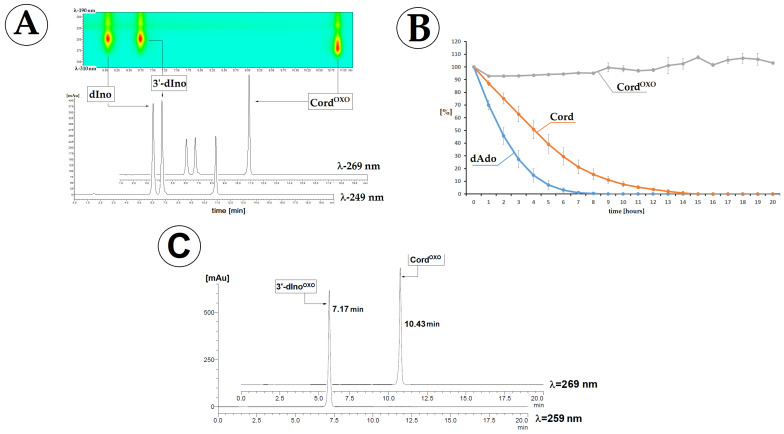
(**A**) RP-HPLC chromatogram profile recorded in the *λ* range of 190–310 nm of a Cord, dAdo, and Cord^OXO^ mixture after 20 h of deamination reaction catalysed by adenosine deaminase (0.001 U). (**B**) Profile of substrate disappearance during a period of 20 h; results obtained by RP-HPLC monitoring. (**C**) Deamination of Cord^OXO^ after 60 min, catalysed (forced) by a 10^5^ times greater concentration of ADA (100 U) than in the case of results exhibited by A and B graphs (0.001 U).

A mixture of dAdo, Cord, and Cord^OXO^ in equal amounts of 7 [OD] (Optical Density) (46.9 nmol) was treated with 5 μg, corresponding to 0.001 U of ADA (3.5.4.4), in 1 mL of HEPES buffer at 37 °C. As shown in Figure 5A,B, after seven and 14 h, dAdo and Cord were completely exhausted, while Cord^OXO^ displayed high resistance (~100%) to ADA under these conditions. The elution times for dIno and 3′-dIno were measured as 5.7 and 6.8 min, respectively. The concentration of ADA increased to 0.5 mg (100 U), forcing the conversion of Cord^OXO^ to 3′-dIno^OXO^. After one hour, no signal corresponding to the substrate was observed. The final product of the above reaction exhibited a *λ_MAX_* of 259 nm and a longer elution time than dIno and 3′-dIno, i.e., 7.17 min (Figure 5C). The elemental composition of the expected product was confirmed by MS spectroscopy analysis in the related spectra (calculated mass 268.2) with [M − H]^−^ = 266.9 and [M + H]^+^ = 269.3.

### 2.3. DFTB Structural Investigation of Adenosine Deaminase and dAdo, Cord and Cord^OXO^ Settled in the Active Enzyme Centre

**Figure 6:**
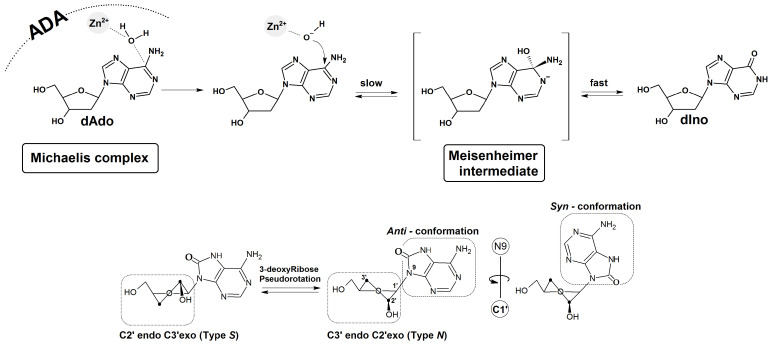

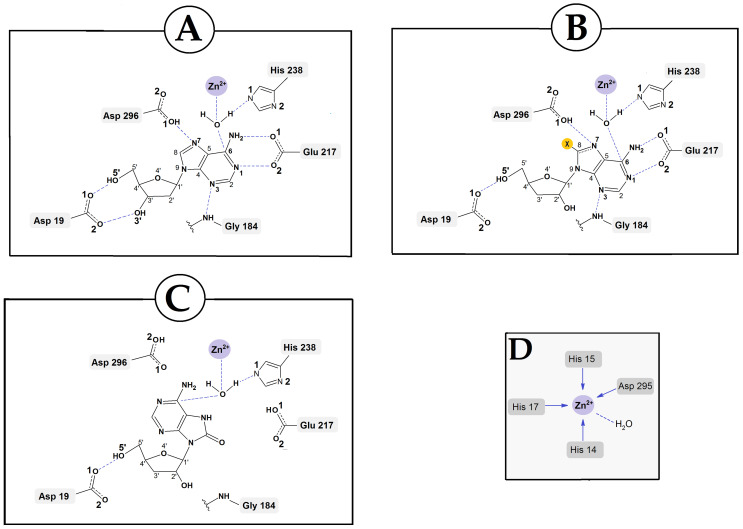
Schematic representation of the adenine nucleoside deamination mechanism by adenosine deaminase (ADA) [29] with graphical representation of the *syn* and *anti* conformers of Cord^OXO^ with 3′-deoxyribose ring geometry, with C2′ endo C3′ exo (Type S) and C3′ endo C2′ exo (Type N) being the most abundant. Catalytic site contacts (Michaelis complex) between adenosine deaminase (1a4l. pdb) and (**A**) dAdo, (**B**) anti-CordOXO (X=O) and Cord (X=H), (**C**) syn Cord^OXO^, obtained at the DFTB/3ob-3-1 level of theory in the aqueous phase (SM12). (**D**) represents the zinc ion (Zn^2+^) coordination in the enzyme active site by His 215, His 217, Asp 295, and “activated” H_2_O.

ADA is a specific enzyme which converts adenine nucleosides to their corresponding hypoxanthine forms in the presence of zinc ions (Zn^2+^) and hydrogen oxide [29]. Nucleic substitution in the aromatic ring is driven by an addition-elimination reaction via the Meisenheimer intermediate, whose lifetime determines the reaction rate (Figure 6) [30].

As shown previously, the *K*_m_ values for dAde and dAdo are approximately 25–35 μM, with catalytic rates of 190 and 176 s^−1^, respectively. These results indicate that the formation of the Michaelis complex between ADA and the substrate is the rate-limiting step of the deamination reaction. The rate of hypoxanthine nucleoside formation, e.g., dIno, is higher than the dissociation rate of the enzyme-substrate complex [31].

To elucidate this phenomenon, Cord^OXO^ was not deemed a suitable substrate for ADA, and a theoretical structural investigation was performed. Because of the system’s complexity, the Density Functional Tight Binding (DFTB) [32] methodology was chosen.

Calculations were performed at the third-order parameterisation level of theory (3ob-3-1) for organic and biological systems in the aqueous phase. The Minnesota Solvation Model 12 (SM12) solvation model described by the Truhlar group was selected [33]. As the starting point of this study, the crystal structure (1a4L. pdb) of the complex between adenosine deaminase and 2′-deoxycoformycin was selected without removing the crystal water molecules [29]. 2′-Deoxycoformycin (2′-dCof) was converted to dAdo, Ado, Cord, and Cord^OXO^, leaving the remaining positions of the atoms unchanged, with subsequent Michaelis complex optimisation. From a thermodynamic perspective, two conformers of Cord^OXO^ are possible (*anti* and *syn*, as shown in Figure 6), which can lead to different biochemical outcomes. Rotation of adenine around the glycosidic bond can give rise to significant changes in the interaction between the adenine moiety and the ADA active site. The sugar ring conformation was selected so as to be similar to that assigned for 2′-deoxycoformicin, i.e., *3′-endo*, *2′-exo* (Type N), typical for ribonucleosides, RNA and DNA A-forms (Figure 6).

It has been previously found that the *syn* conformation is thermodynamically preferred to the *anti*-conformation of Cord^OXO^ [34]. The adenosine deaminase polar active site containing the Zn^2+^ ion is coordinated by His 15, His 17, His 214, Asp 296, Asp 295, and H_2_O molecules, which are activated by His 238. Moreover, previous studies have identified the following hydrogen bonds between the metalloprotein and the substrate as crucial for the deamination reaction (Figure 6 and Table 1: Asp 19:5′ and the 3′ OH group of Ade, Gly184:N3, Glu217:N1 and N6, and Asp296:N7.

The Zn^2+^:H_2_O and H_2_O:C6 distances of the above-mentioned hydrogen bonds found in the optimised structures of ADA with dAdo, Cord, *syn*Cord ^OXO^, and *anti*Cord ^OXO^ are presented in Table 1 and compared with the corresponding ones in the crystal structure of the ADA/2′-dCof Michaelis complex. For all investigated ligands, 2′-dCof (Pentostatin), dAdo, Cord, and *anti*Cord ^OXO^, the lengths of the selected distances were found to be fairly similar. The rotation of adenine molecules around the glycosidic bond and *syn* conformation adoption (the energetically privileged form of Cord ^OXO^) causes a significant elongation of the distance between the C6 atom of adenine and the oxygen atom of H_2_O up to 4.03 Å. This, in turn, prevents any possibility of nucleophilic aromatic substitution via an addition-elimination reaction (Table 1).

## 3. Discussion

Adenosine deaminase was first described almost 100 years ago by Schmidt [35]. This small enzyme (approximately 40 kDa), which belongs to the metalloprotein family, converts adenine to hypoxanthine and related nucleosides/nucleotides via deamination. Therefore, ADA is the main enzyme which regulates the concentration of adenosine derivatives on both sides of the cell membrane [36]. Deficiency of these enzymes leads to severe combined immunodeficiency diseases [37,38]. Due to its abundance and importance, ADA has become the focus of pharmacological research, leading to the discovery and approval of several inhibitors for therapeutic use [39,40,41].

In contrast, the noncanonical nucleoside 3′-deoxyadenosine (Cord) has been identified as a valuable natural compound isolated from the *Cordiceps* fungus. Cord has demonstrated a number of therapeutic effects, including maintaining glucose homeostasis and modulating the immune response, as well as neuroprotective, anticancer, antibiotic, and anti-inflammatory effects. Unfortunately, however, Cord is a suitable substrate for ADA, which converts it to inactive 3′-dIno. It should be noted that cordycepin terminates primer elongation during genetic information replication. Furthermore, due to the lack of a 3′-hydroxyl group, the uptake of Cord through hENTs channels is inefficient [42]. In view of the above, Cord has been administered with ADA inhibitors such as 2′-deoxycoformycin (2′-Conf). As expected, the use of ADA inhibitors leads to an overload of adenosine nucleotides, such as ATP and dATP.

Several adverse effects have been observed, including nausea, vomiting, myelosuppression, fever, and infections [43]. Therefore, this study is the first to focus on the oxidation analogue of Cord, i.e., Cord^OXO^. The above proposition is based on the analogy to dAdo, which, as a purine nucleoside, exhibits a lower ionisation potential than pyrimidines. In contrast, as a triphosphate, dAdo^OXO^ is a suitable substrate for DNA polymerases, showing negligible mutagenicity [23]. Moreover, no specific dAdo^OXO^ glycosylases have been identified. Cord^OXO^ exists in physiological fluids in the *syn* conformation rather than the *anti* conformation typical of canonical nucleosides. Its potential has significant biochemical implications. As shown in this study, however, unlike dAdo and Cord, *syn*Cord^OXO^ is not a suitable substrate for deamination reactions catalysed by adenosine deaminase. Under similar experimental conditions, no Cord^OXO^ conversion to 3′-dIno^OXO^ was observed during RP-HPLC analysis. As Cord^OXO^ might exhibit ADA inhibition properties, experiments were conducted using a mixture of equal amounts of dAdo, Cord, and Cord^OXO^. The obtained results elucidated that under experimental conditions, dAdo and Cord were effectively digested, while Cord^OXO^ was not (Figure 5). This suggests that Cord^OXO^ is a non-suitable substrate for ADA, rather than an inhibitor. However, further studies using specific adenosine deaminase inhibitors, such as pentostatin, are required to confirm this. It should also be noted that increasing the amount of ADA from 0.001 U to 100 U forces the hydrolysis of Cord^OXO^, leading to the formation of 3′-dIno^OXO^, whose structure was confirmed by mass spectroscopic analysis. This phenomenon was elucidated by DFTB structural studies of the ADA Michaelis complex with Cord^OXO^ and a comparison with those of dAdo and Cord. The results of theoretical studies show that rotation of the adenine moiety around the glycosidic bond leads to a lengthening of the distance between hydrogen oxide and C6 carbon of purine of up to 4 Å, which effectively prohibits the deamination process. For the remaining substrates, i.e., dAdo, Cord, and anti-CordOXO, this distance was found to be close to 2.7 Å. The results of the experimental and theoretical studies presented here indicate a delicate balance between the *syn* and *anti*-forms of Cord^OXO^.

## 4. Materials and Methods

### 4.1. Materials

The starting materials/reagents were purchased from Merck (Poznan, Poland) and used directly without any further manipulation. Water was purified using a Milli-Q EQ 7000 Ultrapure Water Purification System (Darmstadt, Germany) before use, and other analytical-grade solvents were used directly. Thin Layer Chromatography (TLC) analyses were performed using silica gel plates 60 F254 from Merck (Poznan, Poland). Chromatographic purification was performed using Merck silica gel 60 (230–400 mesh). Adenosine deaminase from calf intestine (10 mg/2 mL); Roche Diagnostics GmbH, Mannheim, Germany; Ref. 10102105001) was purchased from Merck (Poznan, Poland).

### 4.2. Spectroscopic and Elementary Analysis

The nuclear magnetic resonance (NMR) spectra of ^1^H and ^13^C were recorded in deuterated chloroform (CDCl_3_) and DMSO-*d_6_* on Bruker Avance III spectrometers (600 MHz, Bruker Instruments, Karlsruhe, Germany) with TMS (TetraMethylSilane, Si(CH_3_)_4_) as the internal standard at 600 and 151 MHz, respectively (Laboratory of Molecular Spectroscopy, Faculty of Chemistry, University of Lodz). Chemical shifts are expressed in parts per million (ppm).

Nucleoside concentration was determined using a Varian Cary 1.3E spectrophotometre (Varian, Brunn am Gebirge, Austria) by measuring the maximum absorbance (dAdo: λ = 260 nm, Cord = 260 nm, Cord^OXO^ = 269 nm).

Elemental analyses were performed by the Microanalytical Laboratory of the Centre of Molecular and Macromolecular Studies, Polish Academy of Science, Lodz, Poland, using an ElementarVario MICRO and Elementar Perkin-Elmer PE 2400 CHNS analyser (Perkin Elmer Corp., Norwalk, CT, USA).

High-resolution mass spectrometry (HRMS) was performed using a Waters mass spectrometer, Synapt G2-Si, with an electrospray ionisation (ESI) source and a quadrupole-time-of-flight mass analyser for both positive and negative ion detection modes. The applied parameters for all analyses were as follows: 2.7 kV of capillary voltage; 30 V for cone voltage; 100 °C as source temperature; desolvation nitrogen flow rate, 600 dm^3^/h at a temperature of 350 °C; and nebuliser nitrogen pressure, 6.5 bar. To obtain accurate mass measurements, data were collected in the centroid mode, and all investigated masses were corrected using a leucine encephalin solution as an external reference (during acquisition) with Lock-SprayTM (Waters Corp., Milford, MA, USA), which generated a reference ion at *m*/*z* 554.2615 Da ([M − H]^−^) in the mode ESI and at *m*/*z* 556.2771 Da ([M + H]^+^). The MassLynx 4.1 software (Waters) was used to process the measurement results. HRMS analysis was performed at the Laboratory of Molecular Mass Evaluation, Centre of Molecular and Macromolecular Studies, Polish Academy of Science, Lodz, Poland.

Mass spectra were obtained using a Varian 500-MS IT Mass Spectrometer at the Laboratory of Molecular Spectroscopy, Faculty of Chemistry, University of Lodz. For MS visualisation and signal integration analyses, “Varian Workstation System Control” Version 6.9 with service pack 2 was used.

### 4.3. Procedure for 8-Bromo-3′-Deoxyadenosine Synthesis

Cordycepin (3′-deoxyadenosine, 1.0 g, 0.4 mmol) was suspended in 30 mL of cold water (an ice bath), and 30 mL of saturated bromine water was added dropwise. The reaction mixture was stirred overnight. The progress of the reaction was controlled by TLC analysis CH_3_Cl: CH_3_OH (4:1). The mixture was filtered through Celite and evaporated to dryness under reduced pressure. The product crystallised from cold acetone (20 mL), giving 0.92 g of 8-Bromo-3′-deoxyadenosine with a 70% yield (0.92 g): TLC *R*_f_ = 0.47 (MeOH:CH_3_Cl (1:4); Element anal. calcd. for C_10_H_12_BrN_5_O_3_ in [%] C 36.38, H 3.66, N 21.21; found: C 36.49, H 3.57, N 21.27. ^1^H NMR (600 MHz, DMSO-*d_6_*) δ (in ppm): 8.13 (2H, s, 1H), 7.50 (NH_2_, bs, 2H), 5.75 (1′H, d, 1H, *J^H1′-H2′^* = 4.16 Hz), 5.57 (2′OH, bd, 1H, *J^OH3′-H3′^* = 3.84 Hz), 5.13–5.06 (5′OH, bs, 1H), 5.12–5.07 (4′H, m, 1H), 4.34–4.28 (2′H, m, 1H), 3.62–3.54 (5′H, dd, 1H, *J^H5′-H5″^* = 11.91 Hz, *J^H5′-H4′^* = 3.87 Hz), 3.48–3.41 (5″H, dd, 1H, *J^H5′-H5″^* = 11.91 Hz, *J^H5″-H4′^* = 4.25 Hz), 2.48–2.37 (3′H, m, 1H), 2.02–1.94 (3″H, m, 1H); ^13^C NMR (151 MHz, DMSO-*d_6_*) δ (in ppm): 155.6 (C6), 153.0 (C2), 150.4 (C4), 127.3 (C8), 120.0 (C5), 93.85 (C1′), 80.6 (C4′), 72.39 (C2′), 64.2 (C5′), 35.9 (C3′); HRMS *m*/*z* calculated for C_10_H_11_^79^BrN_5_O_3_ (negative mode, [M − H]^−^) calcd. 328.0045; found: 328.0049, C_10_H_11_^81^BrN_5_O_3_ (negative mode, [M − H]^−^) calcd. 330.0025; found: 330.0028 and C_10_H_13_^79^BrN_5_O_3_ (positive mode, [M + H]^+^) for calcd. 330.0202; found: 330.0207, C_10_H_13_^81^BrN_5_O_3_ (positive mode, [M + H]^+^) for calcd. 332.0181; found: 332.0189.

### 4.4. Procedure A for 7,8-Dihydro-8-Oxo-3′-Deoxyadenosine Synthesis

A solution of sodium benzyloxide was prepared from benzyl alcohol (7.84 g or 7.5 mL, 0.072 mmol) and sodium (0.2 g). To this solution, at room temperature under argon, a solution of 8-bromo-3′-deoxyadenosine (0.5 g, 1.5 mmol) in 10 mL of a benzyl alcohol:DMSO mixture (1:1) was added dropwise with stirring. The progress of the reaction was monitored by TLC analysis to completion (24 h), Rf = 0.43. The reaction mixture was then cooled to ~4 °C (ice bath) and carefully neutralised with a 20% HCl water solution. CH_2_Cl_2_ (50 mL) was added, and the organic layer was separated, washed with a saturated NaHCO_3_ solution (2 × 20 mL), washed once more with water (10 mL), and dried over MgSO_4_. After filtration, the organic layer was evaporated under reduced pressure. Crude 8-benzyloxy-3′-deoxyadenosine was used in the next stage of the synthesis without further purification.

Crude 8-benzyloxy-7,8-dihydro-8-oxo-3′-deoxyadenosine (0.65 g) was dissolved in 50 mL of dry methanol and hydrogenated overnight over a 10% Pd/C catalyst (0.2 g) under increased hydrogen pressure at room temperature. The reaction was monitored by TLC, CHCl_3_:MeOH (4:1). The catalyst was removed by filtration through Celite and subsequently washed twice with 50 mL of hot methanol. The organic solvents were removed under reduced pressure and the residue was purified by column chromatography (on silica gel) in chloroform with a methanol gradient of 0 to 5% to give 0.2 g after two synthetic steps (total yield 50%): TLC *R*_f_ = 0.40, developing system: MeOH:CH_3_Cl (1:4); ^1^H NMR (600 MHz, DMSO-*d_6_*) δ (in ppm): 11.59 (NH_2_, bs, 2H), 8.35 (2H, s, 1H), 5.64 (1′H, d, 1H, *J^H1′-H2′^* = 3.23 Hz), 4.87–4.82 (4′H, m, 1H), 4.26–4.12 (2′H, m, 1H), 3.54–3.49 (5′H, dd, 1H, *J^H5′-H5″^* = 11,37 Hz, *J^H5′-H4′^* = 5.17 Hz), 3.47–3.42 (5″H, dd, 1H, *J^H5′-H5″^* = 11.37 Hz, *J^H5′-H4′^* = 4.74 Hz), 2.42–2.35 (3′H, m, 1H), 1.94–1.87 (3″H, m, 1H); ^13^C NMR (151 MHz, DMSO-*d_6_*) δ (in ppm): 151.20 (C6), 146.6 (C8), 146.3 (C4), 143.4 (C2), 104.4 (C5), 89.7 (C1′), 80.6 (C4′), 72.5 (C2′), 64.1 (C5′), 35.6 (C3′); HRMS *m*/*z* calcd. for C_10_H_12_N_5_O_4_ (negative mode, [M − H]^−^) 266.0889; found: 266.0894, and calcd. for C_10_H_14_N_5_O_4_ (positive mode, [M + H]^+^) 268.1046; found: 268.1047.

### 4.5. Procedure B for 7,8-Dihydro-8-Oxo-3′-Deoxyadenosine Synthesis

The 8-bromo-3′-deoxyadenosine (0.4 g, 1.21 mmol) was suspended in a mixture of 30 mL of water, 0.378 g (0.34 mL, 4.84 mmol) of 2-mercaptoethanol, and 1.23 g (1.7 mL, 10 mmol) of triethylamine. The reaction mixture was refluxed for five hours, and the progress was monitored by TLC (CH_3_Cl:MeOH, 4:1). After the substrate was fully exhausted, the volatile compound was evaporated under reduced pressure. The product was isolated by column chromatography (on silica gel) in chloroform with a methanol gradient of 0 to 5% to give 0.25 g of pure product (total yield 76%).

### 4.6. General Procedure for Adenosine Deaminase Activity—UV Assay

The adenosine deaminase activity assay was performed in a 1 mL reaction mixture of 50 mM HEPES (4-(2-hydroxyethyl)-1-piperazineethanesulfonic acid) buffer, pH = 7.3. An appropriate amount, i.e., 66 nmol, corresponding to 1 [OD], (Optical Density [44]) of the investigated nucleoside, i.e., dAdo, Cord or Cord^OXO^_,_ was dissolved. The reaction was initiated by the addition of an appropriate amount of adenosine deaminase (25 ng), corresponding to 0.005 U. The reaction was monitored by measuring the UV absorbance change at 21 °C in the range of 220–350 nm every five minutes for one hour using a Varian Cary 1.3E spectrophotometre (Varian, Brunn am Gebirge, Austria).

### 4.7. General Procedure for Adenosine Deaminase Activity—RP HPLC Assay

The adenosine deaminase activity assay was performed in a 1 mL reaction mixture of 50 mM HEPES (4-(2-hydroxyethyl)-1-piperazineethanesulfonic acid) buffer at pH 7.3. An appropriate amount (462 nmol, corresponding to 7 [OD]) of the investigated nucleoside, i.e., dAdo, Cord or Cord^OXO^_,_ was added. The reaction was initiated by the addition of an appropriate amount of adenosine deaminase (5 ng), corresponding to 0.001 U, and the reaction was monitored at 37 °C. HPLC spectra with a 190–310 nm detection range were archived every 60 min for 20 h. All analyses were performed in triplicate.

Each high-performance liquid chromatography (HPLC) analysis was performed using a C-18 column, Synergi 4 μm Fusion-RP 80 Å, 250 × 4.6 mm, flow rate 1 mL/min with UV detection in the range of 190–350 nm. The following buffers were used: (A) 0.1 M ammonium acetate (pH~7) and (B) CH_3_CN. The HPLC analytical gradient was as follows: from 0 to 15 min, the concentration of B increased from 0 to 8%, with a subsequent decrease to 0% from 15 to 20 min.

### 4.8. Geometry Optimisation

The starting geometries of 3′-deoxyadenosine, 2′-deoxyadenosine, and 7,8-dihydro-8-oxo-3′-deoxyadenosine were obtained by modifying the 2′-deoxycoformycin (Pentostatine) structure from its complex with adenosine deaminase of 1a4l. pdb [29]; the peptide, water molecules, and Zn^2+^ were left unmodified. The three-dimensional geometries of the Michaelis complexes, i.e., ADA-dAdo, ADA-Cord, ADA-*anti*Cord^OXO^, and ADA-*syn*Cord^OXO^_,_ were optimised to the ground state using the Density Functional Tight Binding (DFTB) methodology [32] with a self-consistent redistribution of Mulliken charge modification (SCC) [45]. The Third-Order Parameterisation for Organic and Biological Systems, with suspended Main Improvements Over the parameter set, noted as 3ob-3-1, was applied [46]. The Minnesota Solvation Model 12 (SM12) was used instead of a periodic box due to structure complexity [33]. The generalised Born/solvent-accessible surface area model (GBSA) for water was applied using a solvent-accessible angular surface grid of 230. The convergence criterion for the SCC-DFTB interaction was set to ≤10^−5^ Hartree. For the theoretical geometry optimisations, the molecular ADF (Amsterdam Density Functional) program suite, version 2023.101 (part of Software for Chemistry & Materials B.V. Amsterdam, The Netherlands) [47].

## 5. Conclusions

Adenosine deaminase (ADA) is one of the most important enzymes in nucleoside metabolism and is present on both sides of the cell membrane. This small protein can exchange the amino group of adenine derivatives (e.g., ATP and dATP) with oxygen atoms, thus effectively inactivating their native properties and maintaining homeostasis. However, ADA can deactivate or reduce the therapeutic molecules built on the adenine-leading structure, which requires their co-administration with an adenosine deaminase inhibitor. 3′-Deoxyadenosine (Cordycepin, Cord) is a promising natural compound with anticancer activity owing to its ability to inhibit primer elongation of lagging strands during the replication of genetic information. Similar to other adenine derivatives, Cord is rapidly deaminated by ADA into inactive 3′-deoxyinosine, which limits its application with pentostatin.

In this study, the stability of 3′-deoxyadenosine (Cord), 7,8-dihydro-8-oxo-3′-deoxyadenosine (Cord^OXO^), and 2′-deoxyadenosine (dAdo) in adenosine deaminase presets was taken into consideration. The study confirms that under the experimental conditions, Cordycepin is digested more slowly than dAdo by amidohydrolase. Furthermore, RP-HLC with UV detection has become a useful analytical technique for process monitoring. A similar usefulness of UV spectroscopy monitoring was noted for pure enzymes.

Comparative studies show that Cord^OXO^ is completely stable under the experimental conditions required for dAdo and Cord conversion to suitable dIno and 3′-dIno. The conversion of Cord^OXO^ to 3′-dIno^OXO^ was observed after a significant increase in the amount of ADA.

The results of the theoretical studies (DFTB) show that after Cord^OXO^ adopts a *syn* conformation, the distance between the hydrogen oxide of ADA and the C6 carbon of purine increases up to 4 Å, which effectively prohibits the deamination process by amidohydrolase. In contrast, in the case of *anti*Cord^OXO^ the distance was similar to that observed for dAdo and Cord, i.e., 2.7 Å.

In conclusion, considering the above, it can be theoretically predicted that Cord^OXO^ could become a valuable pharmacological molecule capable of terminating primer elongation during DNA replication, especially in rapidly proliferating cells, as in the case of cancer. It should be underlined, however, that any therapeutic effect should be achieved without ADA inhibitors, such as pentostatin, to minimise adverse side effects. Therefore, Cord^OXO^ warrants further investigation in order to thoroughly assess its therapeutic potential.

## 6. Further Perspectives and Remarks

DNA damage, such as 7,8-dihydro-2′-deoxyguanosine/adenosine, has been well investigated not only as an isolated lesion but also as part of clustered damage. However, in contrast, scant information can be found in the literature concerning the role of oxidised therapeutics based on an adenine structure. Therefore, the susceptibility of molecules like Cord towards one-electron oxidising processes must be assessed. Moreover, the toxicity of derivatives generated by gamma radiation or the Haber−Weiss reaction catalysed by transition metal activity should also be assessed in the context of normal and cancer (pathological) cell lines. Model studies of deaminase deficiency (in lymphoblastoid cell lines) should be undertaken to investigate how Cord^OXO^ exerts its cytotoxic effects. From the perspective of anticancer therapy, the possibility of Cord^OXO^ migrating through the cell membrane and being subsequently incorporated into the genome sheds light on its therapeutic potential. For this purpose, molecular dynamics should be initially performed in a physiological medium. Additionally, the answer to the above question may result in improved efficacy of combined anticancer treatments, such as radiotherapy and chemotherapy.

## Figures and Tables

**Table 1:** Calculated distances in [Å] present in the catalytic site (Michaelis complex) between adenosine deaminase (1a4l. pdb) and ligands dAdo, *anti*Cord^OXO^, Cord, *syn*Cord ^OXO^, obtained at the DFTB/3ob-3-1 level of theory in the aqueous phase (SM12) and compared with those assigned to the crystal structure of ADA and 2′-dCof (2′-deoxycoformycin, Pentostatin). * Distances from initial crystal structure.

Protein ADA	Atom Number	Ligand				
		2′-dCof	dAdo	Cord	*anti*Cord^OXO^	*syn*Cord^OXO^
Asp 19 O1	O5′	2.80 */2.80	2.95	2.90	2.97	3.34
Asp 19 O2	O3′	2.89 */2.88	3.10	----	----	----
Gly 184 N	N3	3.29 */3.30	3.45	3.40	3.40	6.60
Glu 217 O1	N6	----	3.76	3.70	3.74	6.53
Glu 217 O2	N1	2.79 */2.79	3.14	3.15	3.15	8.74
His 238 N1	O (H_2_O)	3.25 */3.25	2.84	2.84	2.84	2.86
Asp 296 O1	N7	2.76 */2.76	3.15	3.20	3.23	5.48
Zn^2+^	O (H_2_O)	1.84 */1.83	2.12	2.11	2.11	2.15
O (H_2_O)	C6 (8 *)	1.60 */1.60	2.73	2.76	2.72	4.03
His 14 N	Zn^2+^	2.67 */2.67	2.04	2.96	2.04	2.05
His 15 N		2.78 */2.78	2.02	3.34	2.03	2.07
His 17 N		2.73 */2.73	1.98	1.96	1.99	2.00
Asp 295 O		2.38 */2.39	2.81	2.83	2.84	2.82

## Data Availability

Data are contained within the article and Supplementary Materials.

## Supplementary Materials

The following supporting information can be downloaded at: https://www.mdpi.com/article/10.3390/molecules30163377/s1, Figure S1. ^1^H NMR spectrum (600 MHz, DMSO-*d_6_*) of 8-Bromo-3′-deoxyadenosine (Cord^Br^). Figure S2. ^13^C NMR spectrum (151 MHz, DMSO-*d_6_*) of 8-Bromo-3′-deoxyadenosine (Cord^Br^). Figure S3. ^1^H NMR spectrum (600 MHz, DMSO-*d_6_*) of 7,8-dihydro-8-oxo-3′-deoxyadenosine (Cord^OXO^). Figure S4. ^13^C NMR spectrum (151 MHz, DMSO-*d_6_*) of 7,8-dihydro-8-oxo-3′-deoxyadenosine (Cord^OXO^). Figure S5. High-Resolution Mass Spectrum (HRMS ESI) of 8-Bromo-3′-deoxyadenosine in negative ion mode [M − H]^−^, *m*/*z* calculated for isotope ^79^Br, 328.0045; found 328.0049. Figure S6. High-Resolution Mass Spectrum (HRMS ESI) of 8-Bromo-3′-deoxyadenosine in negative ion mode [M − H]^−^, *m*/*z* calculated for isotope ^81^Br, 330.0025; found 330.0028. Figure S7. High-Resolution Mass Spectrum (HRMS ESI) of 8-Bromo-3′-deoxyadenosine in positive ion mode [M + H]^+^, *m*/*z* calculated for isotope ^79^Br, 330.0202; found 330.0207. Figure S8. High-Resolution Mass Spectrum (HRMS ESI) of 8-Bromo-3′-deoxyadenosine in positive ion mode [M + H]^+^, *m*/*z* calculated for isotope ^81^Br, 332.0181; found 332.0189. Figure S9. Mass Spectrum (MS) of 8-benzyloxy-3′-deoxyadenosine sodium salt in positive ion mode [M + H]^+^, *m*/*z* calculated 380.4; found 380.3. Figure S10. High-Resolution Mass Spectrum (HRMS ESI) of 7,8-dihydro-8-oxo-3′-deoxyadenosine (Cord^OXO^) in positive ion mode [M + H]^+^, *m*/*z* 268.1046; found 268.1047. Figure S11. High-Resolution Mass Spectrum (HRMS ESI) of 7,8-dihydro-8-oxo-3′-deoxyadenosine (Cord^OXO^) in negative ion mode [M − H]^−^, *m*/*z* 266.0889; found 266.0894. Figure S12. Mass Spectrum (MS) of 7,8-dihydro-8-oxo-3′-deoxyinosine in negative ion mode [M − H]^−^, *m*/*z* calculated 268.3; found 266.9. Figure S13. Mass Spectrum (MS) of 7,8-dihydro-8-oxo-3′-deoxyinosine in positive ion mode [M + H]+, *m*/*z* calculated 268.3; found 269.3. Table S1. Raw data of nucleoside digestion by adenosine deaminase, monitored by RP-HPLC (λ = 260 nm) analysis. Table S2. Average value, given in percentage [%], and the standard deviation of nucleoside digestion by adenosine deaminase. Optimized *.pdb structures of adenosine deaminase with: 2′-deoxyadenosine (dAdo_ADA.pdb), 2′-deoxycoformycin (dCoformycin_ADA.pdb), 3′-deoxyadenosine (Cord_ADA.pdb), *anti* 8-oxo-3′-deoxyadenosine (CordOXO_Anti_ADA.pdb) and *syn* 8-oxo-3′-deoxyadenosine (CordOXO_Syn_ADA.pdb).
