# Supplementary material for: 8-OXO-Cordycepin Is Not a Suitable Substrate for Adenosine Deaminase-Preliminary Experimental and Theoretical Studies
Source: Molecules. 2025 Aug 14;30(16):3377. doi: 10.3390/molecules30163377 (PMC12388660; doi:10.3390/molecules30163377)
Supplement: Supplementary file 1 [file molecules-30-03377-s001.zip › molecules-3773917-supplementary.pdf]

Supplementary Materials

8-OXO-Cordycepin is not a Suitable Substrate for Adenosine Deaminase: Preliminary Experimental and Theoretical Studies

Boleslaw T. Karwowski

Nucleic Acids Damage Laboratory, Faculty of Pharmacy, Medical University of Lodz, ul. Muszynskiego 1, 90-151 Lodz, Poland; Boleslaw.Karwowski@umed.lodz.pl

Figure S1. ^{1}H NMR spectrum (600 MHz, DMSO-d_6) of 8-Bromo-3'-deoxyadenosine (Cord^{Br}).

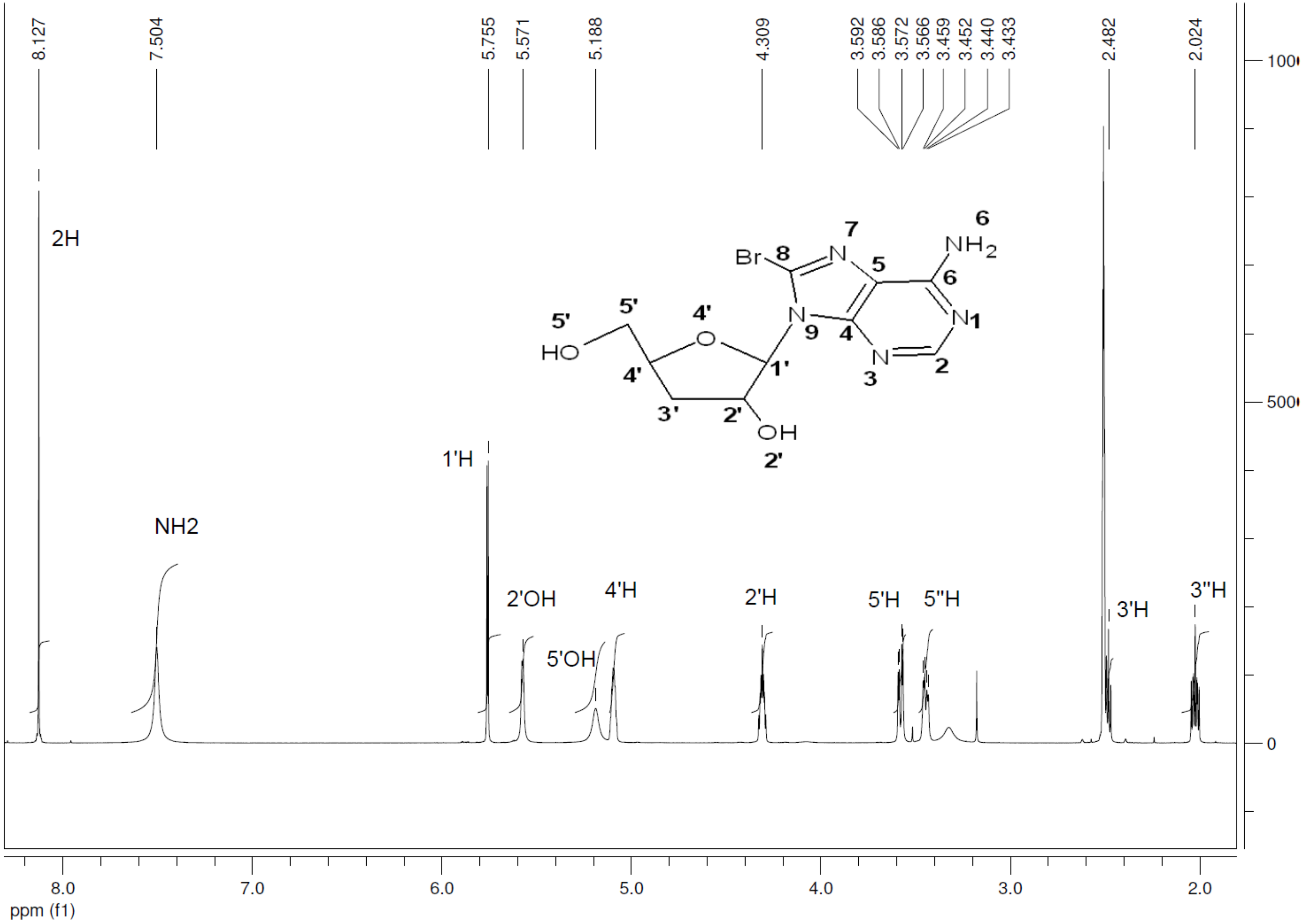

Figure S2. ^{13}C NMR spectrum (151 MHz, DMSO-d_6) of 8-Bromo-3'-deoxyadenosine (Cord^{Br}).

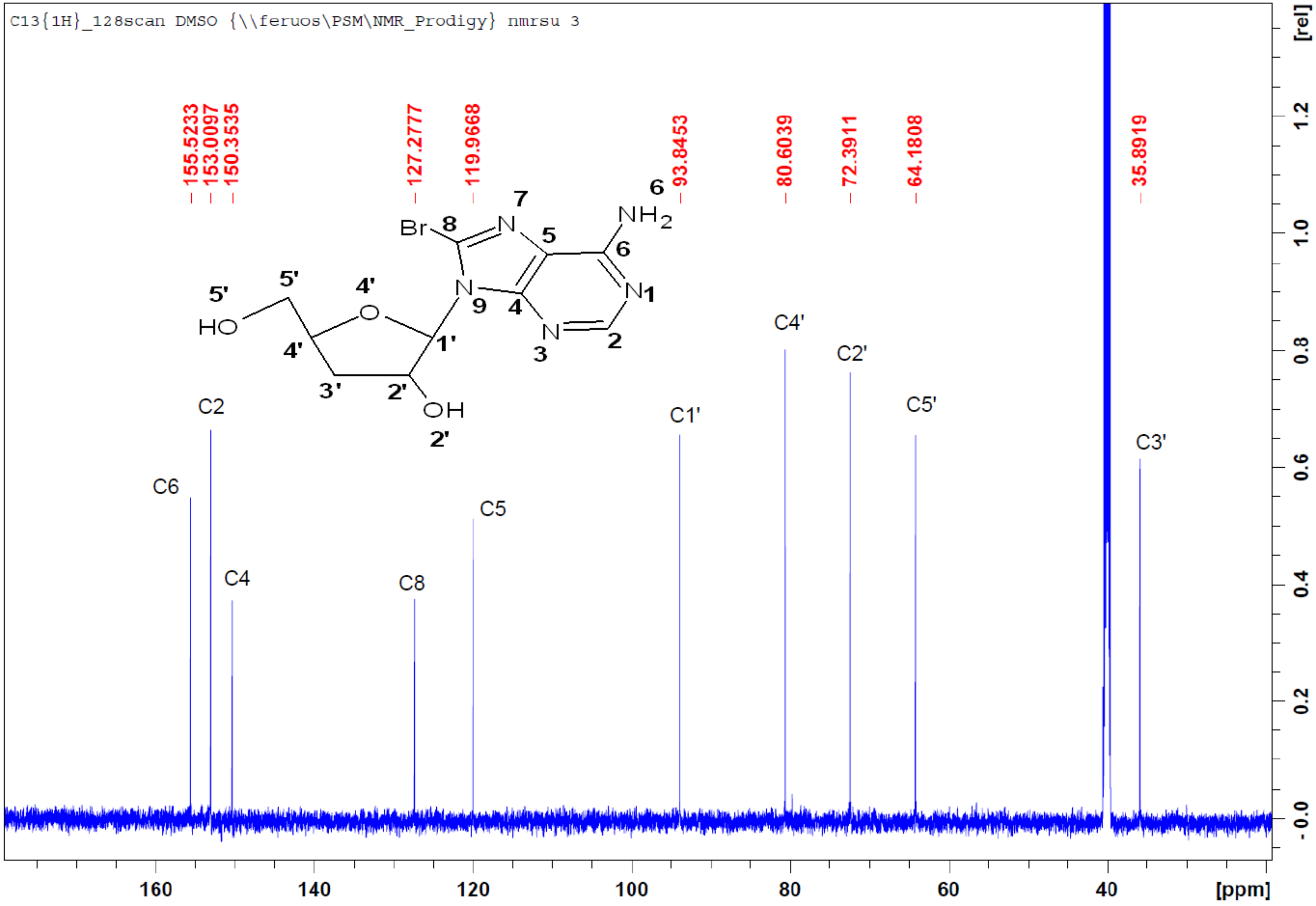

Figure S3. ^{1}H NMR spectrum (600 MHz, DMSO-d_6) of 7,8-dihydro-8-oxo-3'-deoxyadenosine (Cord^{OXO}).

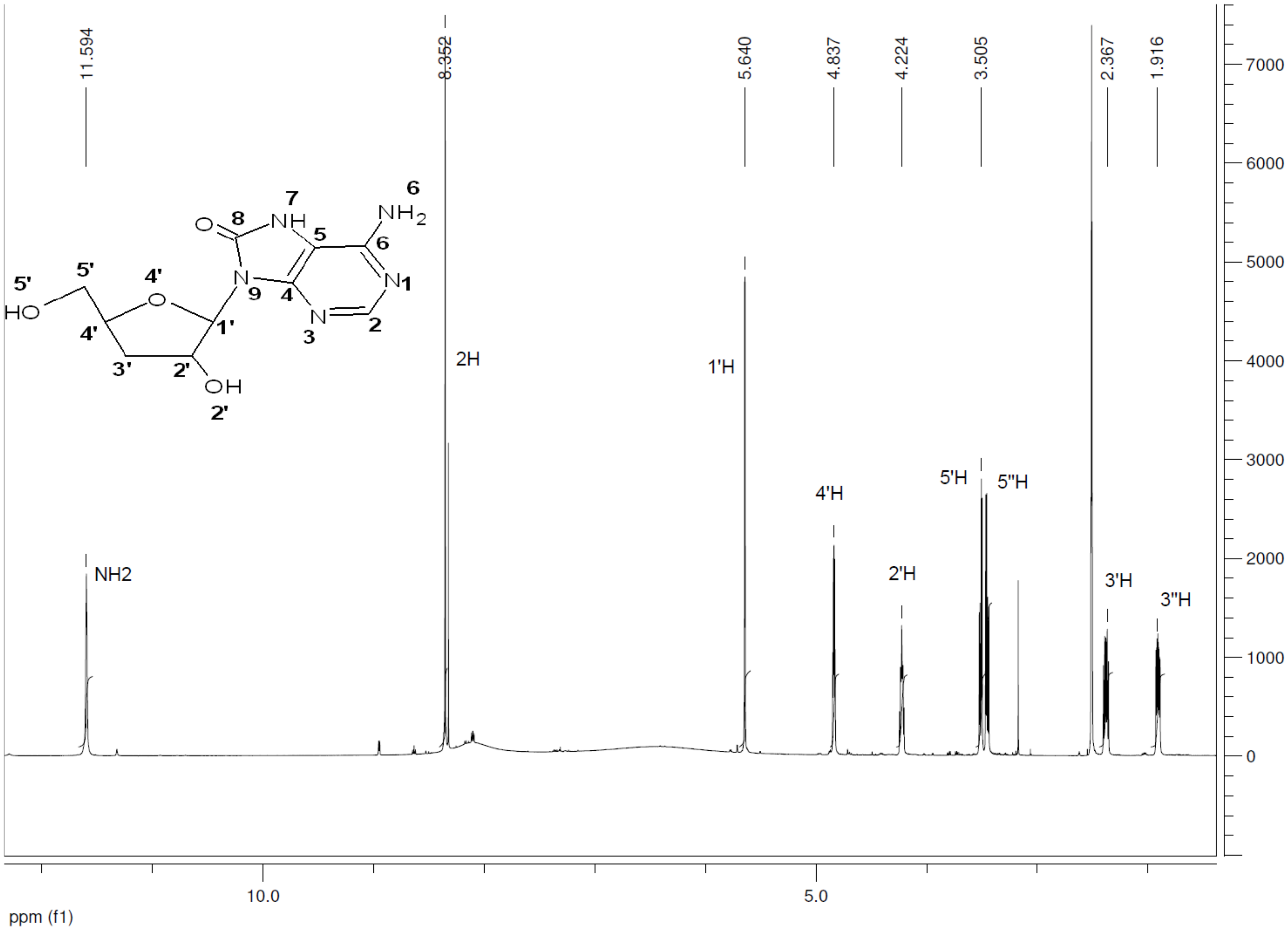

Figure S4. ^{13}C NMR spectrum (151 MHz, DMSO-d_6) of 7,8-dihydro-8-oxo-3′-deoxyadenosine (Cord^{OXO}).

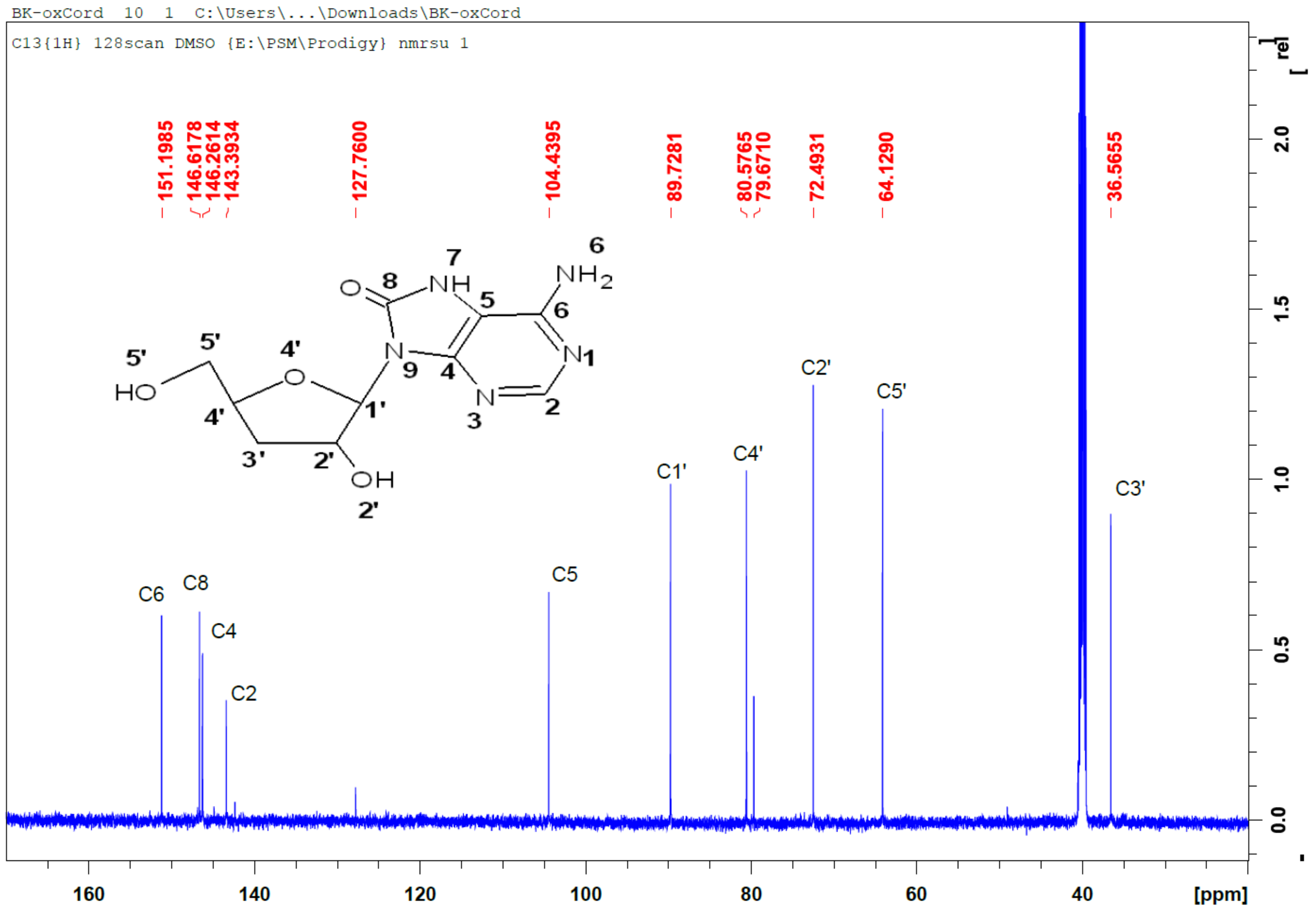

Figure S5. High-Resolution Mass Spectrum Spectra (HRMS ESI) of 8-Bromo-3'-deoxyadenosine in negative ion mode $[M\text{-}H]^-$, *m/z* calculated for isotope ^{79}Br, 328.0045, found 328.0049.

Elemental Composition Report

Page 1

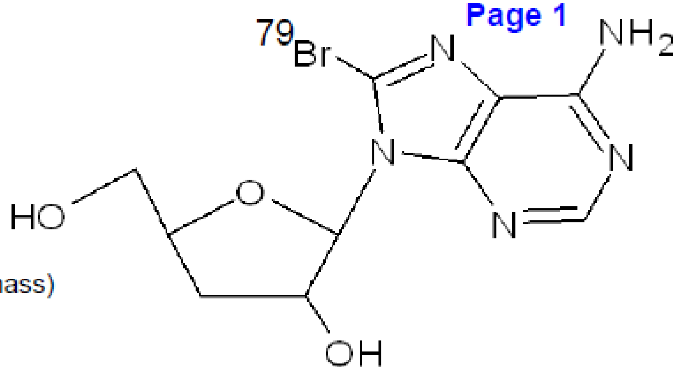

Single Mass Analysis

Tolerance = 5.0 PPM / DBE: min = -1.5, max = 150.0
Element prediction: Off
Number of isotope peaks used for i-FIT = 9

Monoisotopic Mass, Even Electron Ions
758 formula(e) evaluated with 2 results within limits (all results (up to 1000) for each mass)
Elements Used:
C: 0-45 H: 0-60 N: 0-6 O: 0-9 79Br: 0-1 81Br: 0-1

250325_BK_BrCard_A 25 (0.277) Cm (21:32)

TOF MS ES-
1.55e+006

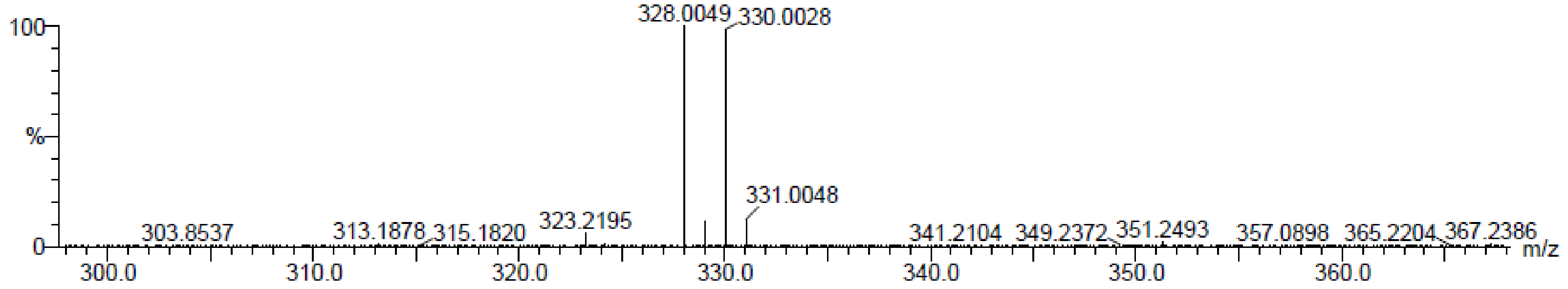

Minimum:				-1.5				
Maximum:		5.0	5.0	150.0				
Mass	Calc. Mass	mDa	PPM	DBE	i-FIT	Norm	Conf(%)	Formula
328.0049	328.0045	0.4	1.2	7.5	690.7	0.620	53.77	C10 H11 N5 O3 79Br
	328.0035	1.4	4.3	22.5	690.8	0.772	46.23	C22 H2 N O3

Figure S6. High-Resolution Mass Spectrum (HRMS ESI) of 8-Bromo-3'-deoxyadenosine in negative ion mode $[M\text{-}H]^-$, *m/z* calculated for isotope ^{81}Br, 330.0025, found 330.0028.

Elemental Composition Report

Page 1

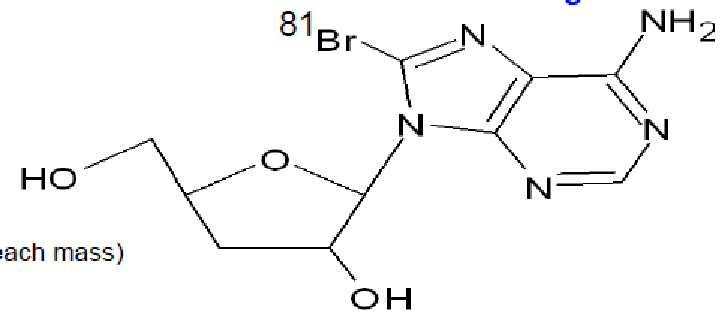

Single Mass Analysis

Tolerance = 5.0 PPM / DBE: min = -1.5, max = 150.0
Element prediction: Off
Number of isotope peaks used for i-FIT = 9

Monoisotopic Mass, Even Electron Ions
766 formula(e) evaluated with 2 results within limits (all results (up to 1000) for each mass)
Elements Used:
C: 0-45 H: 0-60 N: 0-6 O: 0-9 79Br: 0-1 81Br: 0-1

250325_BK_BrCard_A 25 (0.277) Cm (21:32)

TOF MS ES-
1.55e+006

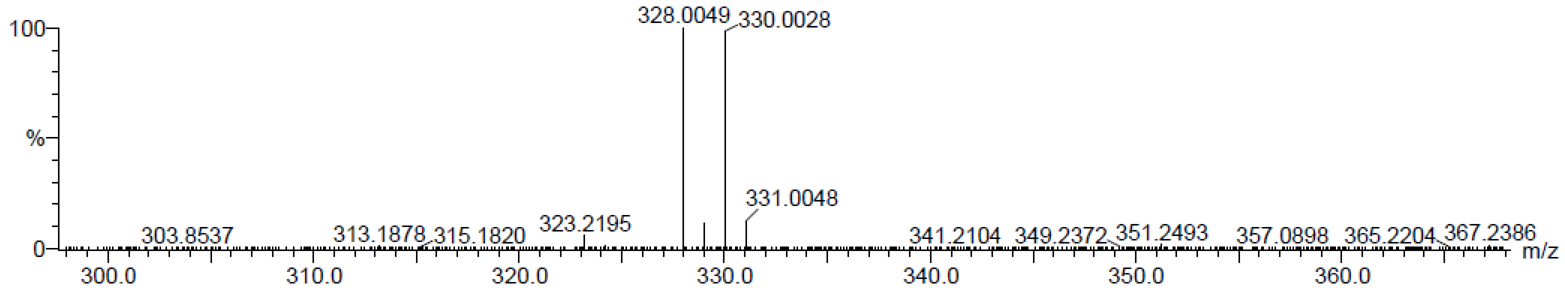

		mDa	PPM	DBE				
Minimum:				-1.5				
Maximum:		5.0	5.0	150.0				

Mass	Calc. Mass	mDa	PPM	DBE	i-FIT	Norm	Conf(%)	Formula
330.0028	330.0025	0.3	0.9	7.5	681.0	0.000	99.99	C10 H11 N5 O3 81Br
	330.0039	-1.1	-3.3	17.5	690.8	9.836	0.01	C18 H4 N O6

Figure S7. High-Resolution Mass Spectrum (HRMS ESI) of 8-Bromo-3'-deoxyadenosine in positive ion mode $[M+H]^+$, *m/z* calculated for isotope ^{79}Br, 330.0202, found 330.0207.

Elemental Composition Report

Page 1

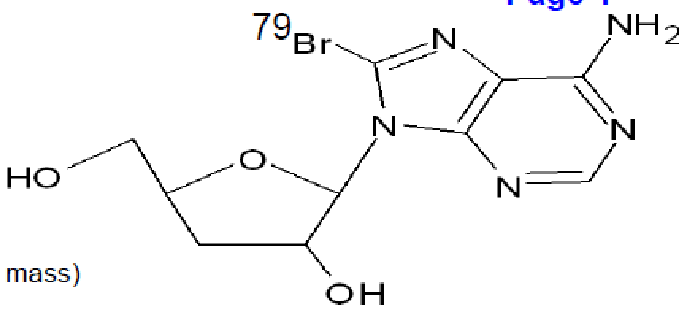

Single Mass Analysis

Tolerance = 5.0 PPM / DBE: min = -1.5, max = 150.0
Element prediction: Off
Number of isotope peaks used for i-FIT = 9

Monoisotopic Mass, Even Electron Ions
765 formula(e) evaluated with 2 results within limits (all results (up to 1000) for each mass)
Elements Used:
C: 0-45 H: 0-60 N: 0-6 O: 0-9 79Br: 0-1 81Br: 0-1

250325_BK_BrCard_p_A 29 (0.311) Cm (29:33)

TOF MS ES+
6.81e+006

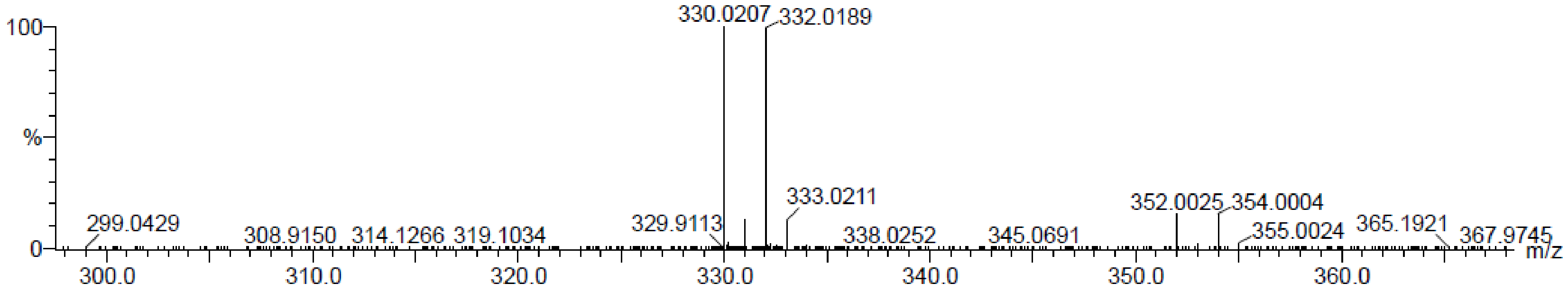

		mDa	PPM	DBE				
Minimum:				-1.5				
Maximum:		5.0	5.0	150.0				
Mass	Calc. Mass	mDa	PPM	DBE	i-FIT	Norm	Conf(%)	Formula
330.0207	330.0202	0.5	1.5	6.5	1162.9	0.627	53.39	C10 H13 N5 O3 79Br
	330.0191	1.6	4.8	21.5	1163.1	0.763	46.61	C22 H4 N O3

Figure S8. High-Resolution Mass Spectrum (HRMS ESI) of 8-Bromo-3’-deoxyadenosine in positive ion mode $[M+H]^+$, *m/z* calculated for isotope ^{81}Br, 332.0181, found 332.0189.

Elemental Composition Report

Page 1

Single Mass Analysis

Tolerance = 5.0 PPM / DBE: min = -1.5, max = 150.0
Element prediction: Off
Number of isotope peaks used for i-FIT = 9

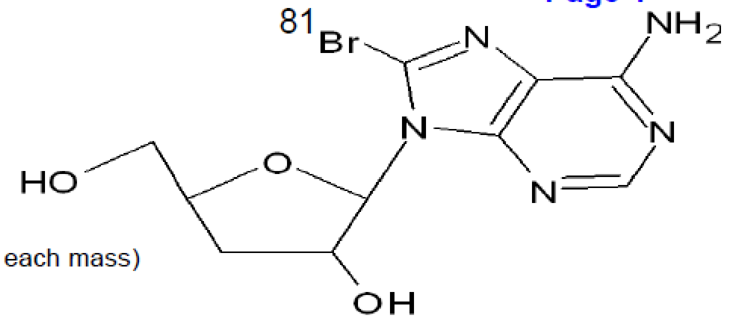

Monoisotopic Mass, Even Electron Ions
778 formula(e) evaluated with 2 results within limits (all results (up to 1000) for each mass)
Elements Used:
C: 0-45 H: 0-60 N: 0-6 O: 0-9 79Br: 0-1 81Br: 0-1

250325_BK_BrCard_p_A 29 (0.311) Cm (29:33)

TOF MS ES+
6.81e+006

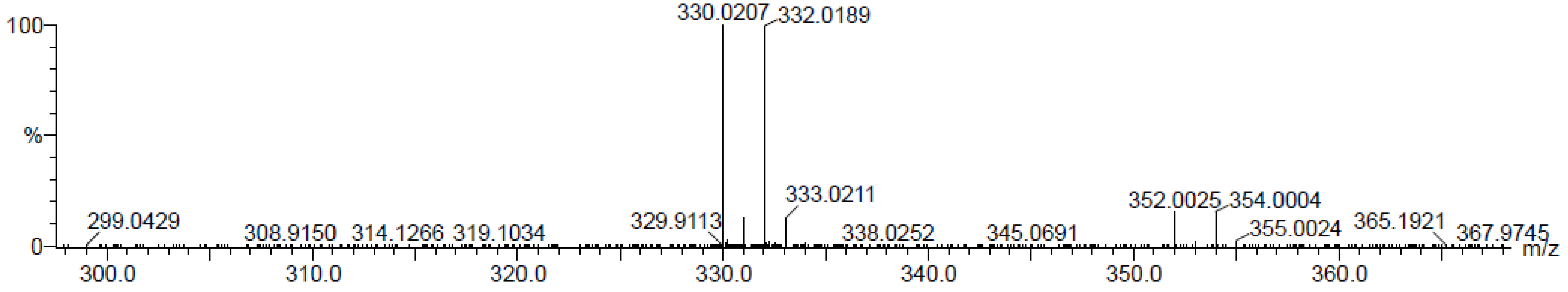

Minimum:				-1.5				
Maximum:		5.0	5.0	150.0				

Mass	Calc. Mass	mDa	PPM	DBE	i-FIT	Norm	Conf(%)	Formula
332.0189	332.0181	0.8	2.4	6.5	819.6	0.000	100.00	C10 H13 N5 O3 81Br
	332.0195	-0.6	-1.8	16.5	830.9	11.280	0.00	C18 H6 N O6

Figure S9. Mass Spectrum (MS) of 8-benzyloxy-3’-deoxyadenosine sodium salt in positive ion mode $[M+H]^+$, *m/z* calculated 380.4, found 380.3.

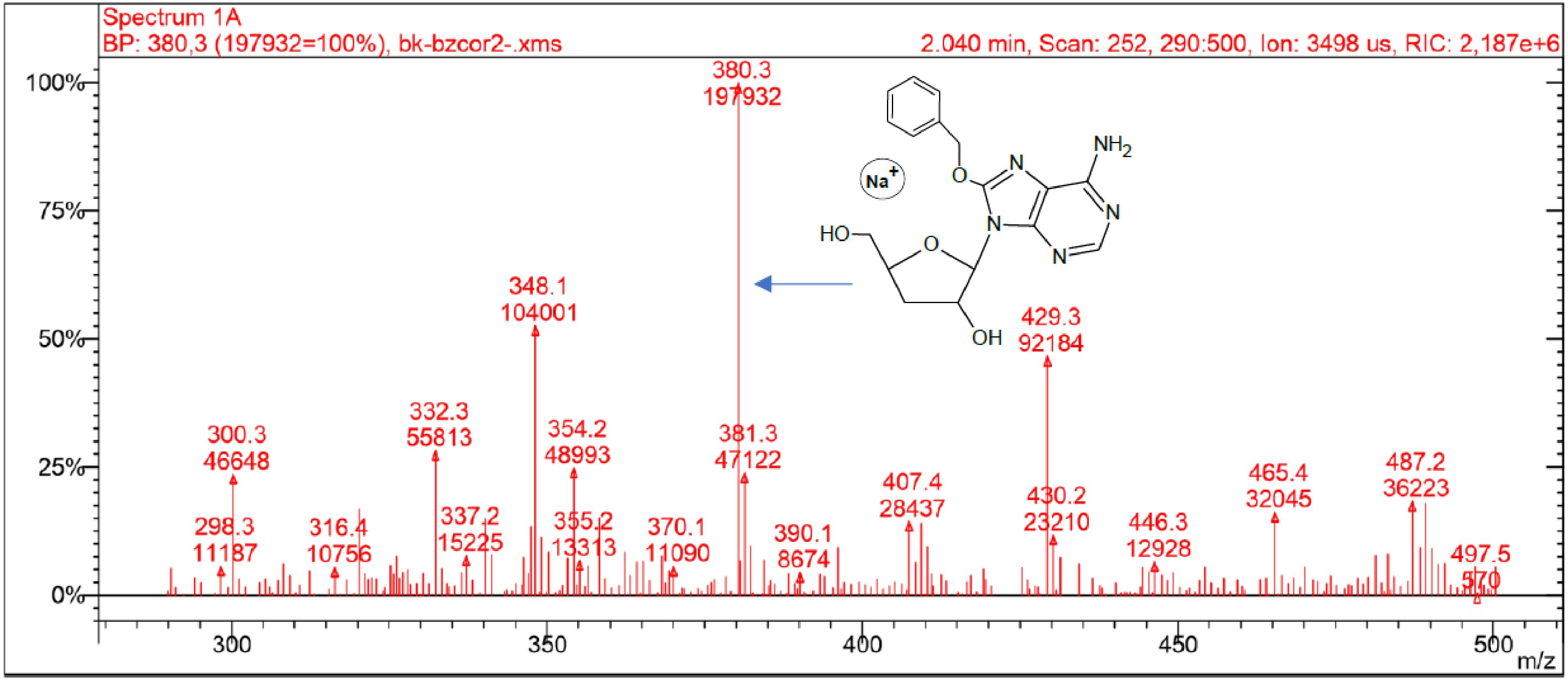

Figure S10. High-Resolution Mass Spectrum (HRMS ESI) of 7,8-dihydro-8-oxo-3’-deoxyadenosine (Cord^{OXO}) in positive ion mode [M+H]$^{+}$, *m/z* 268.1046; found: 268.1047.

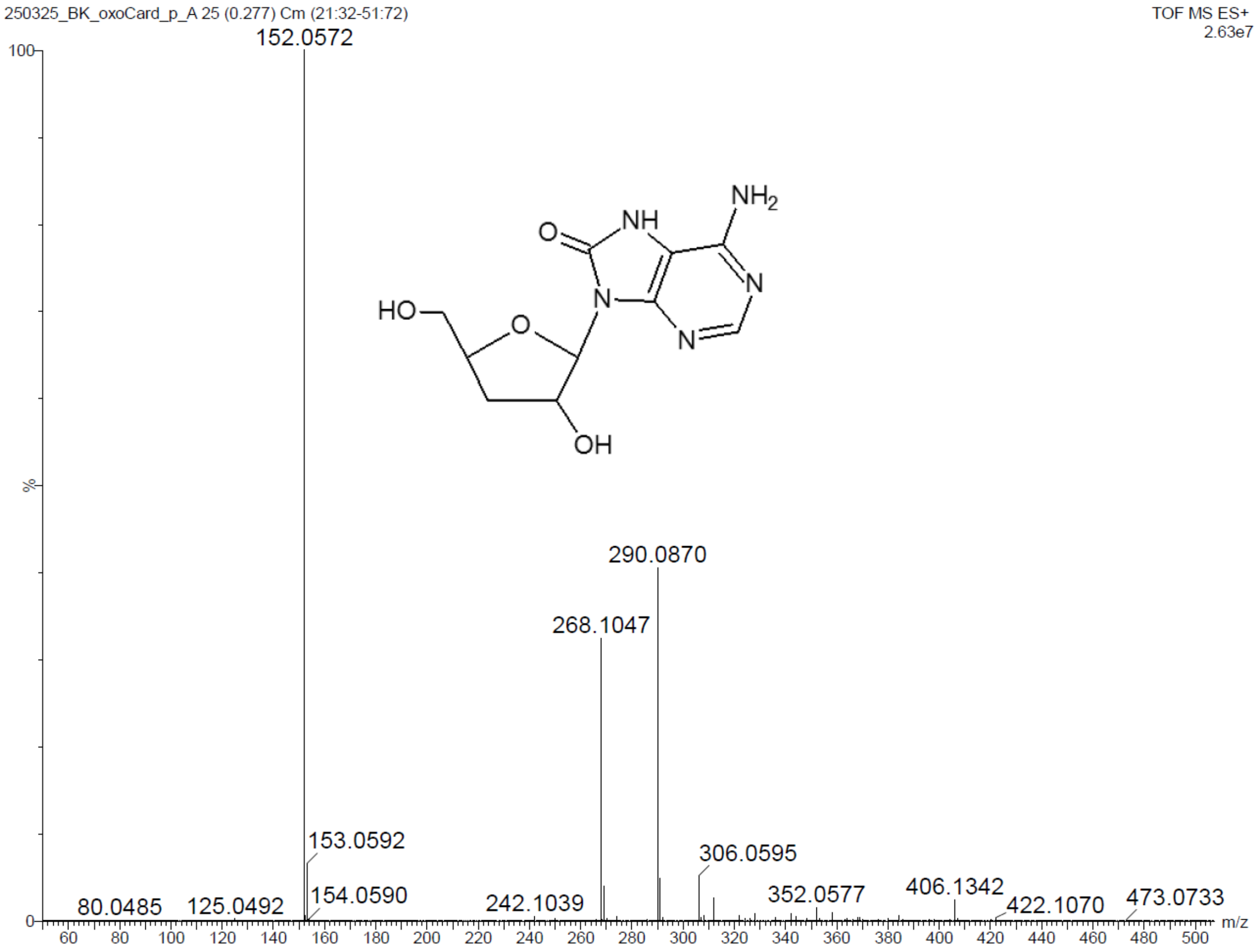

Figure S11. High-Resolution Mass Spectrum (HRMS ESI) of 7,8-dihydro-8-oxo-3'-deoxyadenosine (Cord^{OXO}) in negative ion mode $[M-H]^-$, *m/z* 266.0889; found: 266.0894.

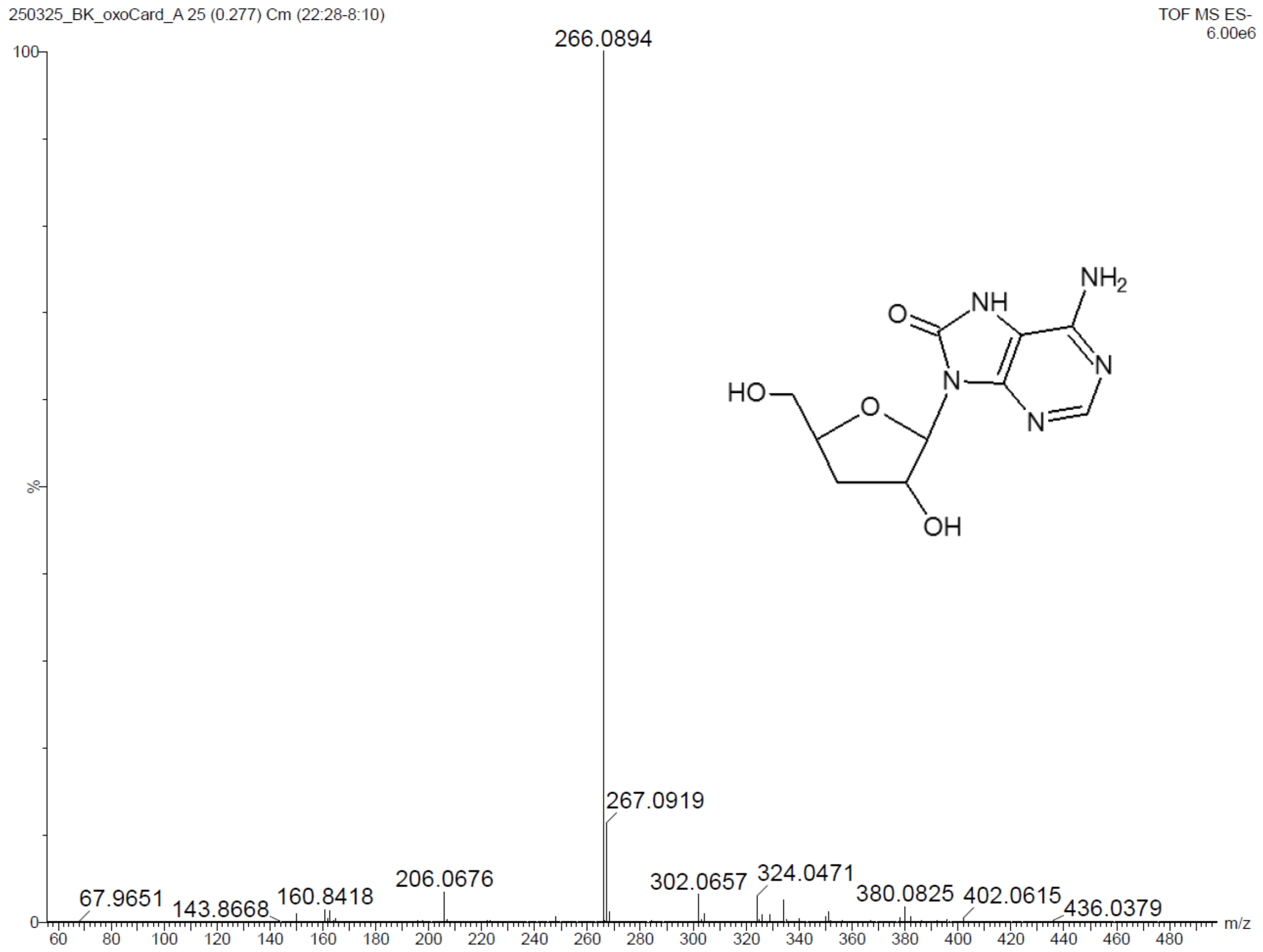

Figure S12. Mass Spectrum (MS) of 7,8-dihydro-8-oxo-3’-deoxyinosine in negative ion mode $[M-H]^-$, *m/z* found 266.9, calculated molecular mass 268.3.

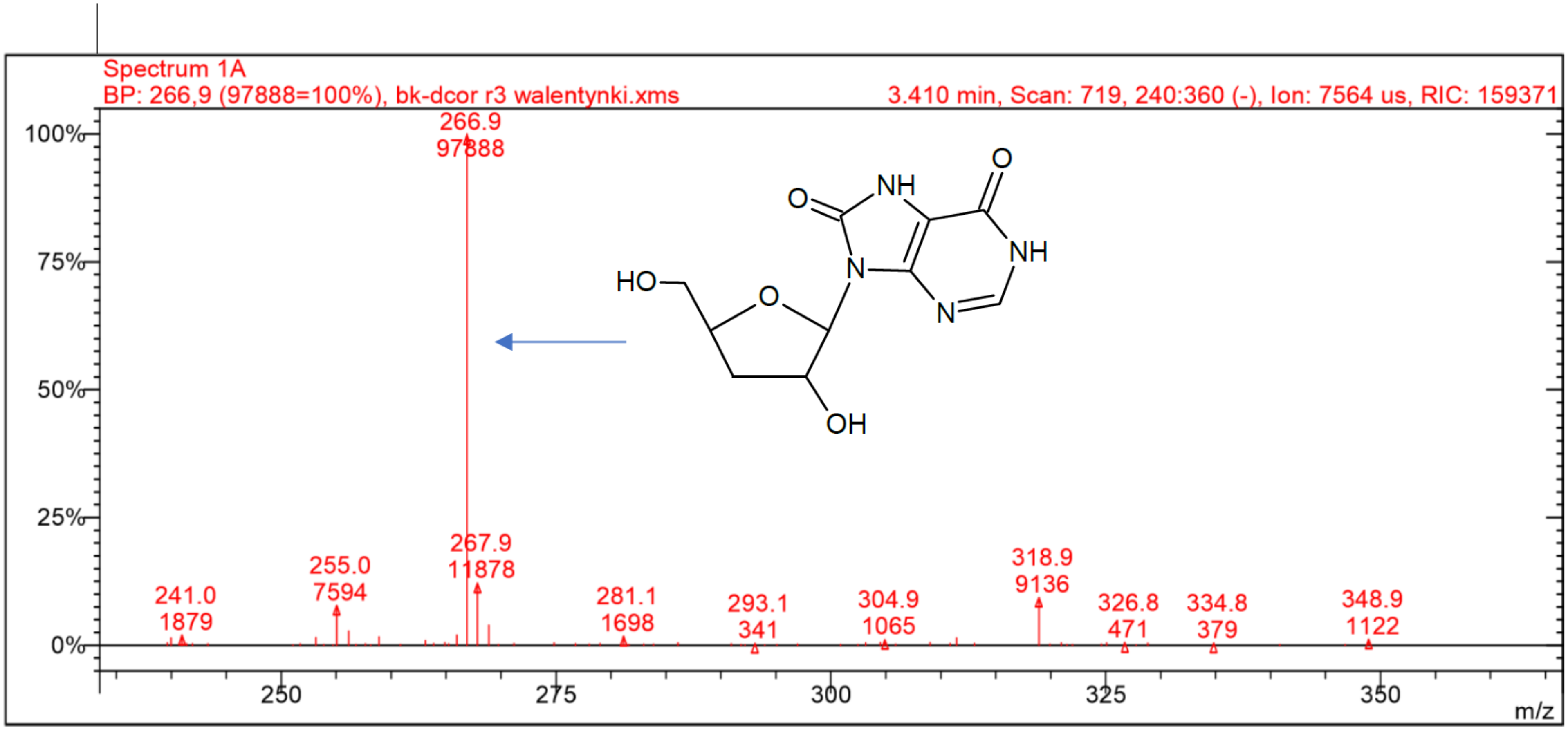

Figure S13. Mass Spectrum (MS) of 7,8-dihydro-8-oxo-3'-deoxyinosine in positive ion mode $[M+H]^+$, *m/z* found 269.3, calculated molecular mass 268.3.

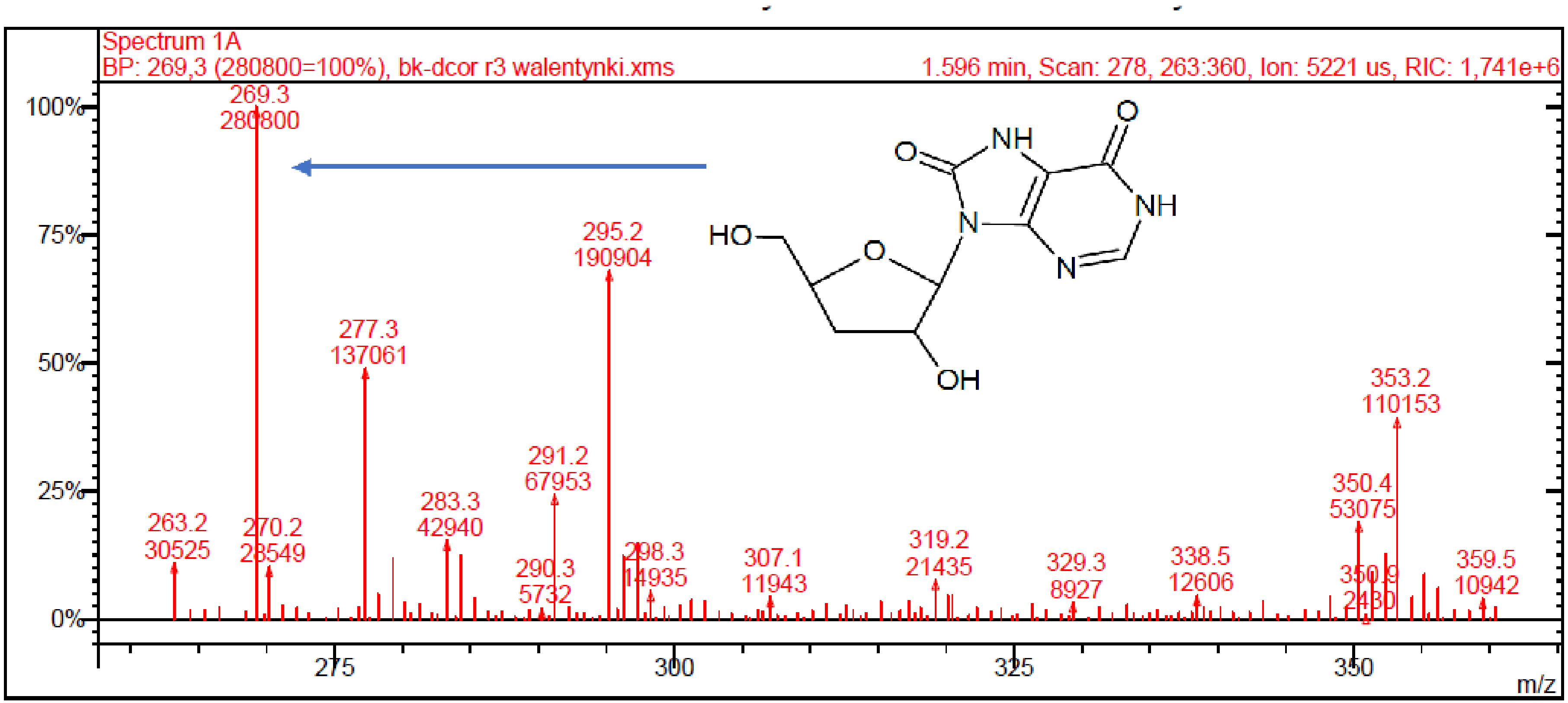

Table S1. Raw data of nucleoside digestion by adenosine deaminase, monitored by RP-HPLC (λ=260 nm) analysis.

The calculated percentage nucleoside digestion by adenosine deaminase, monitored by RP-HPLC analysis at λ=260 nm.									
Time in hours	Repetition 1			Repetition 1			Repetition 1		
	dAdo	Cord	Cord^{OXO}	dAdo	Cord	Cord^{OXO}	dAdo	Cord	Cord^{OXO}
0	100.00	100.00	100.00	100.00	100.00	100.00	100.00	100.00	100.00
1	71.14	88.29	93.43	65.79	84.69	93.11	73.06	88.24	91.87
2	47.59	76.68	93.13	38.52	70.24	93.75	51.04	78.55	91.66
3	29.18	65.38	92.99	19.72	55.87	94.01	32.95	67.57	92.36
4	16.06	53.38	93.57	8.71	42.52	94.03	19.33	56.46	92.74
5	7.82	41.99	93.96	3.44	30.97	94.98	10.07	45.07	93.58
6	3.35	31.51	94.72	1.23	22.03	95.27	4.64	35.22	93.38
7	1.26	22.88	95.32	0.00	15.30	96.15	1.87	25.80	94.44
8	0.53	16.56	94.58	0.00	10.71	96.54	0.68	18.95	94.50
9	0.00	12.04	100.27	0.00	7.85	102.72	0.00	13.24	95.68
10	0.00	8.40	101.15	0.00	5.26	98.29	0.00	9.39	96.10
11	0.00	5.68	95.76	0.00	3.69	98.39	0.00	6.35	96.77
12	0.00	3.74	96.56	0.00	2.66	99.02	0.00	4.63	97.23
13	0.00	2.56	97.13	0.00	0.00	108.75	0.00	3.25	97.74
14	0.00	0.00	106.55	0.00	0.00	102.53	0.00	2.25	98.41
15	0.00	0.00	106.40	0.00	0.00	108.88	0.00	0.00	107.67
16	0.00	0.00	100.26	0.00	0.00	103.25	0.00	0.00	101.38
17	0.00	0.00	102.97	0.00	0.00	104.94	0.00	0.00	108.24
18	0.00	0.00	107.21	0.00	0.00	110.60	0.00	0.00	102.69
19	0.00	0.00	103.05	0.00	0.00	111.59	0.00	0.00	103.39
20	0.00	0.00	103.12	0.00	0.00	103.29	0.00	0.00	103.50
The assigned area under the curve (peak) corresponds to the nucleoside digested by adenosine deaminase. Monitored by RP-HPLC analysis at λ=260nm									
Time in hours	Repetition 1			Repetition 1			Repetition 1		
	dAdo	Cord	Cord^{OXO}	dAdo	Cord	Cord^{OXO}	dAdo	Cord	Cord^{OXO}
0	4275474	4216263	2840524	4214495	4140622	2867702	4080001	3876591	2737579
1	2812625	3570929	2644781	2998087	3655613	2679282	2980656	3420749	2515078
2	1646882	2961605	2663002	2005848	3175149	2670650	2082258	3045135	2509185
3	842999	2355814	2670314	1229986	2707078	2666719	1344307	2619222	2528531
4	372466	1792614	2670974	677040	2210282	2683368	788655	2188558	2538929
5	147050	1305874	2697936	329666	1738782	2694412	410877	1747187	2561732
6	52779	928789	2706206	141038	1304802	2716172	189342	1365350	2556245
7	0	645225	2731221	53188	947175	2733622	76118	1000244	2585396
8	0	451512	2742150	22224	685875	2712325	27597	734673	2587121
9	0	330778	2917816	0	498350	2875424	0	513287	2619247
10	0	221957	2791992	0	347610	2900612	0	363986	2630880
11	0	155642	2794730	0	235389	2746065	0	246079	2649174
12	0	112078	2812590	0	154752	2769167	0	179638	2661686

13	0	0	3088950	0	106154	2785321	0	125843	2675639
14	0	0	2912525	0	0	3055530	0	87117	2694007
15	0	0	3092633	0	0	3051124	0	0	2947649
16	0	0	2932743	0	0	2875236	0	0	2775389
17	0	0	2980948	0	0	2952888	0	0	2963133
18	0	0	3141715	0	0	3074352	0	0	2811130
19	0	0	3169822	0	0	2955088	0	0	2830517
20	0	0	2934090	0	0	2957147	0		2833383

Table S2. Average value, given in percentage [%] and the standard deviation of nucleoside digestion by adenosine deaminase.

Time in hours	The average value, given in percentage [%] of nucleoside digestion by adenosine deaminase			The standard deviation		
	dAdo	Cord	Cord^{OXO}	dAdo	Cord	Cord^{OXO}
0	100.00	100.00	100.00	0.00	0.00	0.00
1	69.99	87.07	92.80	3.77	2.06	0.82
2	45.72	75.16	92.85	6.47	4.36	1.08
3	27.28	62.94	93.12	6.82	6.22	0.83
4	14.70	50.78	93.45	5.44	7.32	0.65
5	7.11	39.35	94.17	3.37	7.41	0.73
6	3.07	29.59	94.45	1.72	6.80	0.97
7	1.04	21.33	95.31	0.95	5.42	0.86
8	0.40	15.41	95.21	0.36	4.24	1.15
9	0.00	11.04	99.56	0.00	2.83	3.58
10	0.00	7.68	98.51	0.00	2.15	2.53
11	0.00	5.24	96.97	0.00	1.38	1.33
12	0.00	3.68	97.60	0.00	0.99	1.27
13	0.00	1.94	101.20	0.00	1.71	6.54
14	0.00	0.75	102.50	0.00	1.30	4.07
15	0.00	0.00	107.65	0.00	0.00	1.24
16	0.00	0.00	101.63	0.00	0.00	1.51
17	0.00	0.00	105.38	0.00	0.00	2.66
18	0.00	0.00	106.83	0.00	0.00	3.97
19	0.00	0.00	106.01	0.00	0.00	4.84
20	0.00	0.00	103.30	0.00	0.00	0.19
